# Global research trends in catheter ablation and surgical treatment of atrial fibrillation: A bibliometric analysis and science mapping

**DOI:** 10.3389/fsurg.2022.1048454

**Published:** 2023-01-06

**Authors:** Xiang Gao, Kai Liu, Xinke Zhao, Xinfang Lv, Xue Wu, Chunzhen Ren, Qilin Chen, Yingdong Li

**Affiliations:** ^1^ Gansu University of Chinese Medicine, Lanzhou, China; ^2^ Affiliated Hospital of Gansu University of Chinese Medicine, Lanzhou, China

**Keywords:** atrial fibrillation, catheter ablation, surgical treatment, bibliometric analysis, science mapping analysis, citation analysis

## Abstract

**Introduction:**

To evaluate the global research results of the catheter ablation and surgical treatment of atrial fibrillation in the past 40 years by bibliometrics, and to explore the hotspots and prospects for future development.

**Methods:**

Relevant literatures were selected from the Web of Science Core Collection. VOSviewer 1.6.17, SciMAT 1.1.04, and CiteSpace 5.8.R1 were used to analyze the data objectively, deeply and comprehensively.

**Results:**

As of July 14, 2021, 11,437 studies for the catheter ablation and surgical treatment of atrial fibrillation have been identified from 1980 to 2021. The *Journal of Cardiovascular Electrophysiology* and *Circulation* respectively ranked first in terms of the number of publications and the number of co-citations. A total of 6,631 institutions from 90 countries participated in the study, with USA leading the way with 3,789 documents. Cryoablation, atrial fibrosis, substrate modification, minimally invasive and access surgery will still be the research focus and frontier in the next few years.

**Conclusions:**

The publication information for the catheter ablation and surgical treatment of atrial fibrillation were reviewed, including country, institution, author, journal publications, and so on. Developed countries had the advantage in this research areas, and cooperation with low-income countries should be improved. The former research hotspots in the field of catheter ablation and surgical treatment of atrial fibrillation were analyzed, and the future research direction was predicted.

## Introduction

Atrial fibrillation (AF) is the most common type of arrhythmia (1, 2). AF is characterized by irregular fibrillation of atrial muscle fibers and loss of effective mechanical contraction when the atria are not coordinated with pulse conduction. Symptoms include palpitations, dizziness, difficulty breathing, fatigue, and decreased exercise capacity (3). There are multiple adverse outcomes, including stroke, heart failure, and dementia (4, 5). The prevalence and incidence of AF increased significantly year by year, and were related to geography, age, and gender (6, 7). Due to the widespread and severe nature of AF, it imposes a huge economic burden on the public health and medical systems in both developed and developing countries. AF accounted for about 1% of the UK National Health Service's budget (8), and $16 to $26 billion has been spent on AF health care in the USA per year (9).

Before the 1980s, left atrial isolation, His bundle dissection and other surgical treatments for AF were limited and could not be effectively promoted (10). Since the Cox-Maze Procedure performed by Cox JL in 1987 opened a new era for the surgical treatment of AF (11), many studies based on this research have sprung up. The Cox-Maze Procedure had been modified to overcome many of these limitations, and the third generation has become the gold standard for AF surgery (12). In recent years, the fourth generation Cox-Maze Procedure which focuses on the use of new energy to ablate, has become a research focus due to its advantages of relatively simple operation, short time, and high safety (13). The ablation methods are constantly improved and optimized, including ablation route, amount of ablation energy, and innovation of ablation instruments. With the revolution of minimally invasive and the help of endoscopic surgery, the development of AF ablation is getting closer to minimally invasive and interventional (14).

In recent years, the research for the catheter ablation and surgical treatment of AF has get more attention, and it is time to conduct a bibliometric analysis of the global publications in this filed. Bibliometric analysis provides a statistical and visible method for assessing the status and trends of a particular research ﬁeld, so that providing ideas and directions for future research (15, 16). This study would analyze the current status and frontier of research in this field, and predict the future trend of scientific research through bibliometric analysis of relevant literature for the catheter ablation and surgical treatment of AF. Meanwhile, this study can help supporters and decision makers to allocate resources and researchers to identify sub-research areas and partners.

## Methods

### Date source and search strategy

The Web of Science Core Collection (WoSCC) was selected as the source for data retrieval, as it provides a comprehensive and standardized data set for reference, and it has been widely used in bibliometric analyses (17). Our research group has developed a comprehensive and highly sensitive retrieval strategy in combination with Boolean logic operators. Search strategy was: TS = (((atrial OR auricular* OR atrium) AND fibrillat*) AND ((catheter OR transcatheter OR percutaneous OR transcutaneous OR surgical) AND (ablat* OR isolat*) OR maze)). We conducted a search by using the WoSCC only including SCI-EXPANDED on the same day on July 14, 2021 to avoid bias caused by database updating. No time or language limitation. We also restricted the document types to article, review, and early access.

The above results were exported to EndNote ×8, and then two researchers in our group independently screened all the retrieved literatures by reading the title, abstract and full text if necessary. After the initial screening, the two researchers cross-checked, and the third researcher participated in resolving differences if necessary, and finally reached a consensus on the inclusion of the documents.

### Statistical analysis

We extracted and summarized the following data: title, publication year, authors, country of authors, sources of journal, impact factor (IF) of journal [The IF of journals was obtained from the 2020 Journal Citation Reports (Clarivate Analytics, 2020)], institutions of authors, co-citation references, co-citation authors, co-citation journals, and keywords. In the process of summarizing the data, we reclassified documents from England, Northern Ireland, Scotland and Wales to the United Kingdom (UK) and documents from Hong Kong, Macau and Taiwan to China (18). Microsoft Excel 2019 was used to make stacked bar chart and analyze the publication trends. The polynomial model (Order number was six) was applied to forecast the growth of publications in the following year. In order to analyze the data more objectively, deeply and comprehensively, we used three bibliometric analysis software VOSviewer 1.6.17, SciMAT 1.1.04 and CiteSpace 5.8.R1 to analyze the data from different perspectives.

VOSviewer, developed by the Netherlands’ Leiden University, is used to extract and analyze the keyword co-occurrence, co-cited and co-authorship information, such as author, institution, reference et al. The author, institution, country collaboration and keyword co-occurrence were visually analyzed by making a network map in VOSviewer 1.6.17 (19). SciMAT (Science Mapping Analysis software Tool) is an open source science mapping software tool developed by University of Granada (20). We used SciMAT to study the incoming and outgoing keywords, and describe the thematic and conceptual evolution for the catheter ablation and surgical treatment of AF. According to the amount of literature, the publications were divided into four consecutive periods: 1991–2005, 2006–2010, 2011–2015, and 2016–2021. Configuration setted in SciMAT was performed as follows: words as the unit of analysis (author and source); 3, 4, 5, 5 as the threshold of data frequency reduction in each period; co-occurrence as the matrix form; 4, 5, 7, 6 as the threshold of data network reduction in each period; equivalence index as the similarity measure to normalize the network; and the simple centers algorithm as the clustering algorithm. CiteSpace is a Java-based application for progressive knowledge domain visualization, it reveals the dynamics in scientific literature as well as visualizing and analyzing trends and patterns in each research field (21). Bursts are defined as a characteristic, which are cited frequently over a period. We used version 5.8.R1 to detect bursts for co-occurrence items, such as authors, institutions, keywords, and co-cited references. We also made a dual-map overlay of journals using CiteSpace.

## Results

### Annual publications and growth forecast

**Figure 1:**
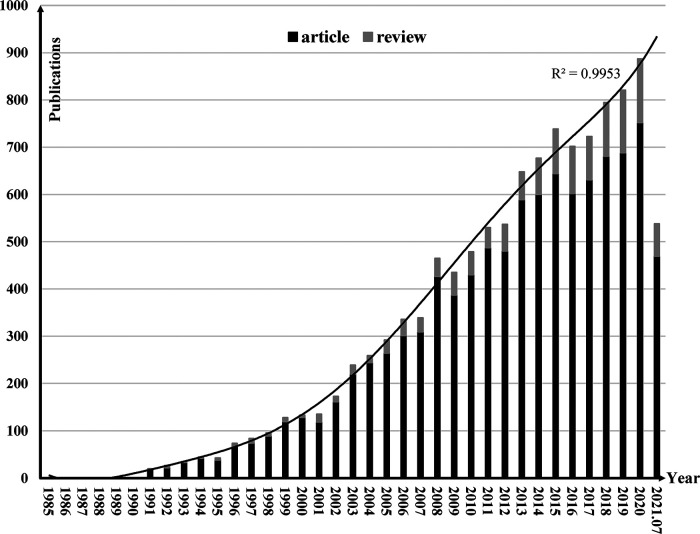
Annual distribution of publications and growth forecast for the catheter ablation and surgical treatment of atrial fibrillation.

The number of reports that were returned in the search included all documents was 12,737. After screening, a total of 11,437 documents were included in the further analysis. Among the 11,437 selected publications, there were 1,230 reviews (11.54%) and original articles (88.46%), which accounted for a larger proportion. Of all published documents, the first for the catheter ablation and surgical treatment of AF was published in 1985 and was written by Sharma AD (22), followed by only one each in 1988 and 1990 over the next five years. Since the 1990s, some attention has been paid to the research in this field gradually. The number of papers published has been increasing slowly every year, and in 1999, more than 100 documents were published. In the 21st century, the number of studies published every year has been more than 100 with steady growth, exceeding 250 in 2004 and 500 in 2011. With the increase of care to the treatment of AF, the number of studies has increased rapidly in the past decade. From 2011 to July 2021, there were 7,597 studies (66.43%) in total. The annual growth rate was negative except in 2016, and the rest were positive every year. As shown in Figure 1, it could be predicted that research in this field will grow more rapidly in the future, and the number of documents published in 2021 was expected to be around 930.

### Co-occurrence keywords and burst keywords

Keywords reflect the core theme and main content of papers, therefore they can provide a reasonable description of research hotspots (23). A total of 13,106 keywords from 11,437 documents were extracted, with the total frequency occurrence of 118,843 times. Table 1 showed the top 20 keywords related to the catheter ablation and surgical treatment of AF. The occurrences of radiofrequency ablation, pulmonary vein isolation, cryoballoon ablation and arrhythmia were the highest, which indicated that they were key to the catheter ablation and surgical treatment of AF.

**Table 1:** Top 20 keywords for the catheter ablation and surgical treatment of atrial fibrillation.

Rank	keyword	Occurrences	Rank	keyword	Occurrences
1	Atrial fibrillation	8,253	11	Risk	1,042
2	Catheter ablation	6,655	12	Arrhythmia	1,014
3	Radiofrequency ablation	3,458	13	Recurrence	817
4	Pulmonary vein isolation	2,641	14	Stroke	798
5	Ablation	2,019	15	Surgical-treatment	796
6	Fibrillation	1,351	16	Impact	745
7	Pulmonary vein	1,177	17	Safety	735
8	Efficacy	1,146	18	Conduction	716
9	Management	1,146	19	Mechanism	709
10	Follow-up	1,114	20	Cryoballoon ablation	703

**Figure 2:**
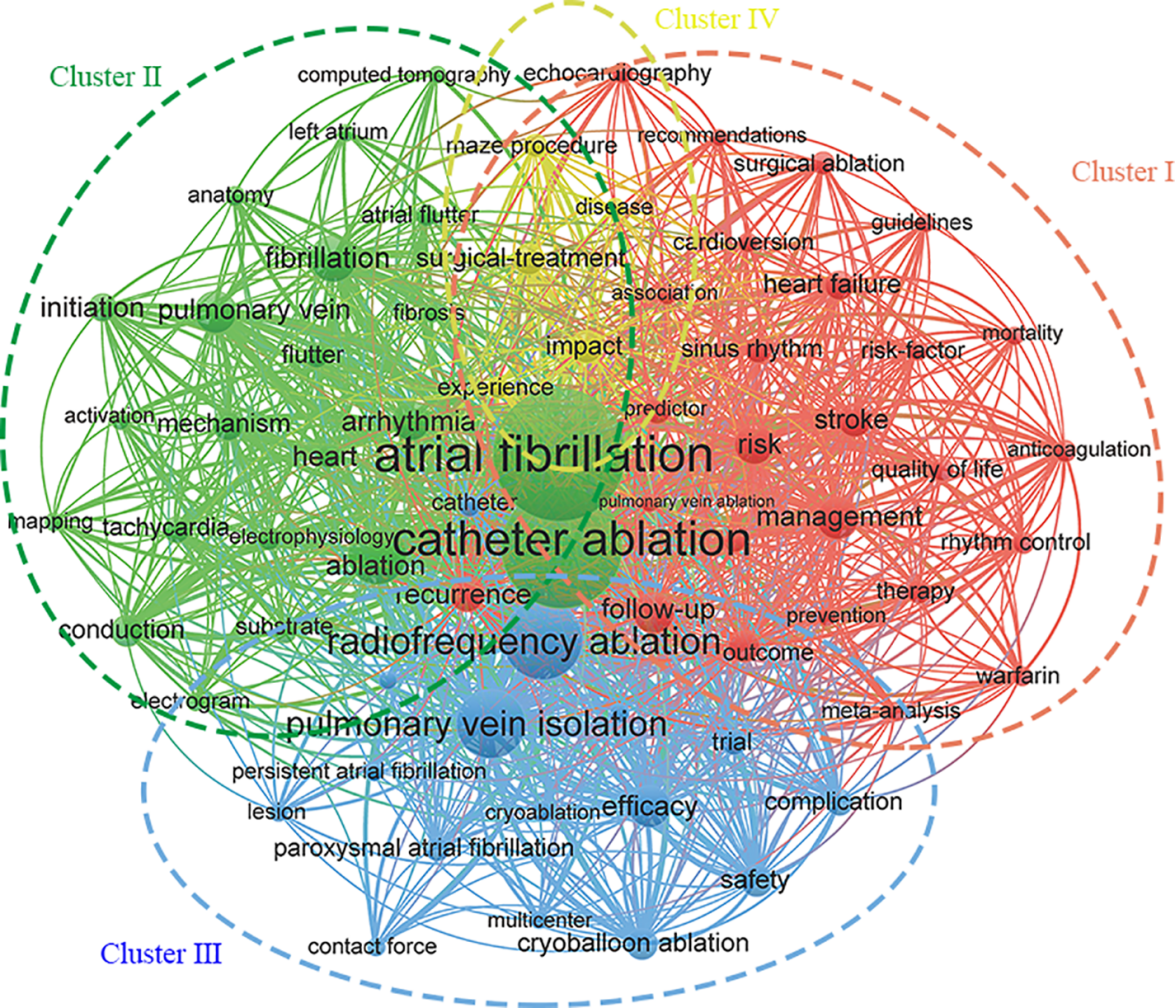
Network map of keywords for the catheter ablation and surgical treatment of atrial fibrillation.

Figure 2 showed the keyword cluster analysis of 250 or more times, and a total of 69 keywords were clustered into four categories. In cluster I with red color, the high frequency keywords were management,stroke, recurrence, risk, predictor, prevalence, therapy, impact, etc. In cluster II with green color, the keywords with higher frequency were catheter ablation, atrial fibrillation, ablation of pulmonary vein, conduction, fibrillation, mechanism, etc. In cluster III with blue color, the key words with high frequency were radiofrequency ablation, pulmonary vein isolation, cryoballoon ablation, contact force, cryoablation, etc. In cluster IV with yellow color, the key words with high frequency were maze procedure and sugical-treatment.

Keywords bursts detected by CiteSpace were shown in Supplementary Appendix A Figure A1. In order to have a clearer understanding of the cutting-edge topics and emerging trends in recent years, the time span was set as 2010–2021, and the minimum duration was set as four. A total of 65 strongly cited keywords were detected. Early keywords (since 2010) were circumferential ablation, complex fractionated atrial electroarar, parkinson white syndrome, congestive heart failure, acute myocardial infarction, etc. Recent bursts (since 2017) were mainly 2nd generationcryoballoon, substrate modification, atrial fibrosis, etc. The longest lasting keyword was acute myocardial infarction and fractionated electrogram, which lasted seven years each. The Burst Strength of atrium (Burst Strength = 11.22), task force (Burst strength = 14.61), heart rhythm association (Burst strength = 10.14), and 2nd generation cryoballoon (Burst strength = 19.2) were both over 10.00. Among them, 2nd generation cryoballoon has the highest Burst strength.

### Theme evolution

**Figure 3:**
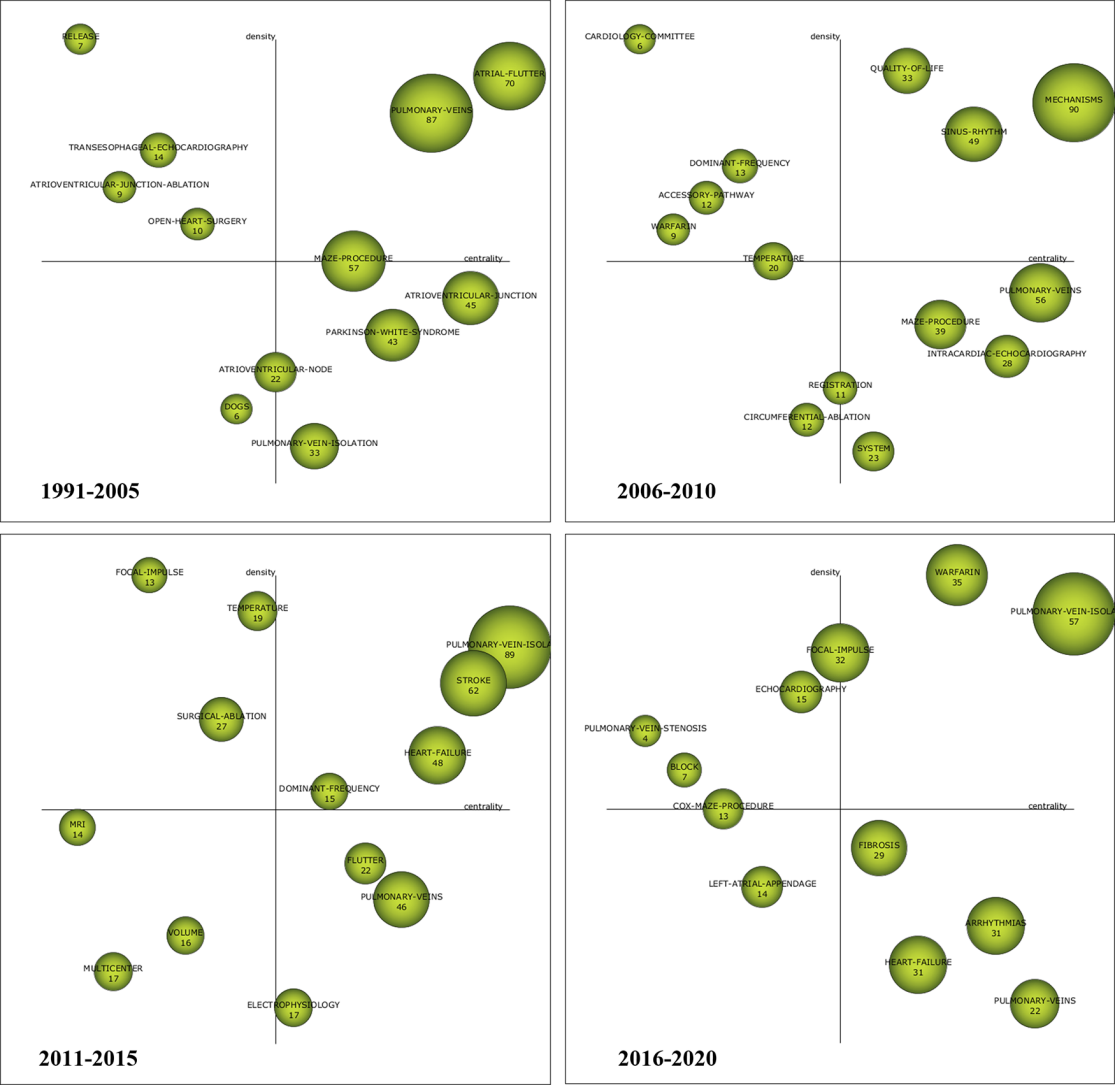
Strategic diagrams for catheter ablation and surgical treatment of atrial fibrillation.

In the strategic diagrams, four different quadrants could be distinguished based on their positions on the map. The upper-right quadrant presents a high density and a strong centrality signifying the most developed themes for the research area studied. The lower-right quadrant includes those keywords that were in the central position but not mature, and had great developmental potential.Themes in the upper-left quadrant have highly developed and isolated themes. The themes of this quadrant have low density and low centrality, mainly representing either emerging or disappearing themes. The size of nodes in the strategic diagrams were proportional to the H-index involved in each words. Figure 3 shows the distribution and changes of motor themes in four different periods. The motor themes of the first period (1991–2005) including atrial flutter, pulmonary veins, and maze procedure. In the second period (2006–2010), researches shifted to mechnisms, sinus rhythm, and quality of life. In the third period (2011–2015), attention was given to pulmonary vein isolation, stroke, heart failure, and dominant frequency. In the last period (2016–2021), pulmonary vein isolation and warfarin had dominated.

**Figure 4:**
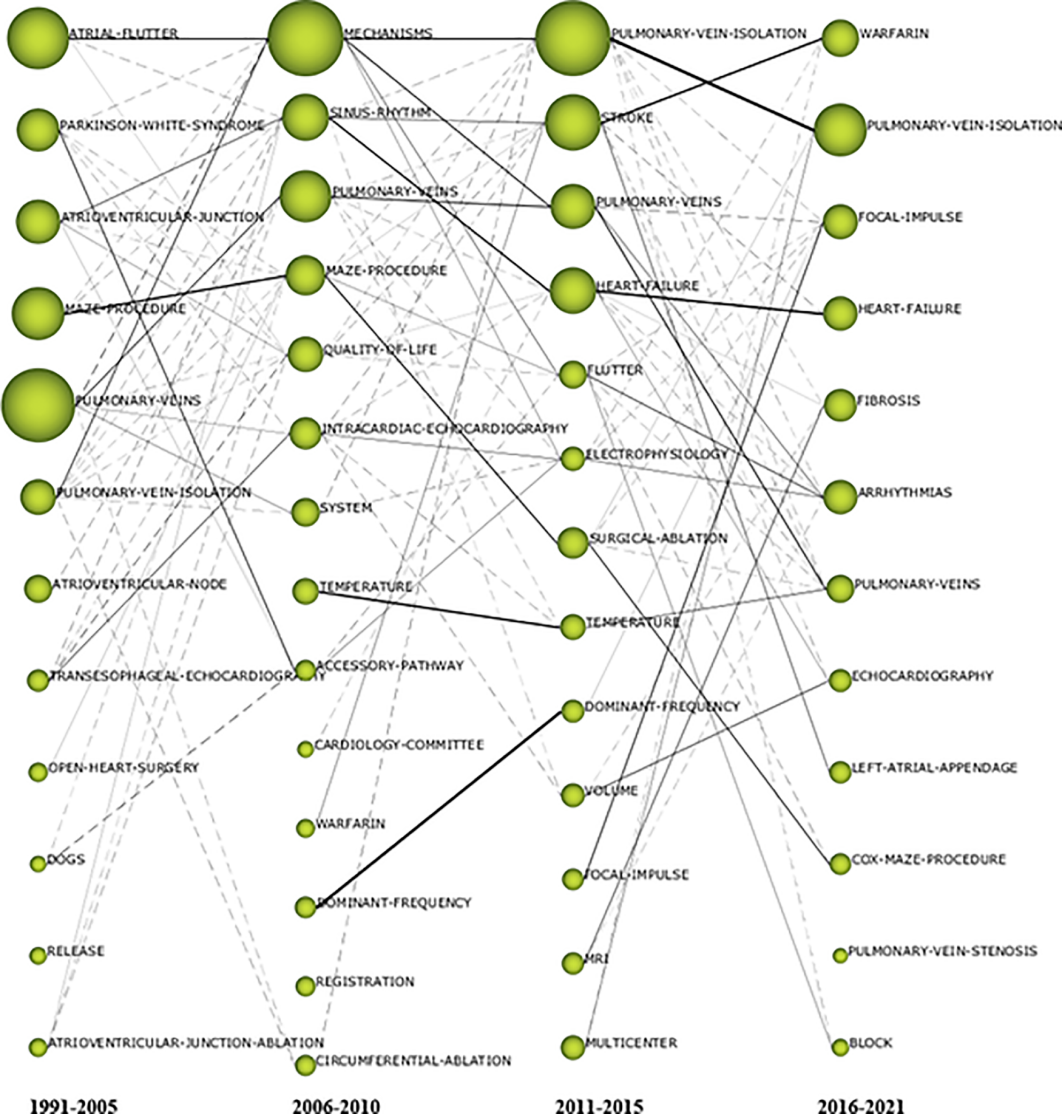
The thematic evolution map for the catheter ablation and surgical treatment of atrial fibrillation.

In the evolution map shown in Figure 4, the nodes represent the clustering of themes in a certain period, the size of nodes is proportional to the H-index associated with each cluster. The solid line represents the linked clustering themes sharing the main analysis units, which indicate that the two themes are persistent and represent the evolution direction of the mainstream. The dotted line indicates the themes sharing elements that were not the main analysis units, representing the evolution direction of tributaries. The thicker the connection of the two theme clusters, the higher their correlation strength and the stronger the evolutionary ability. The isolated node represents the theme that appeared only in a certain period and had no relationship with the theme of the previous and later periods. These isolated nodes reflected, to some extent, the new themes. We can also observe the relationship and trend of changes between keywords in various periods from the perspective of the time axis in Figure 4. The research on the mechanism of AF has greatly promoted the related research of pulmonary vein isolation (ATRIAL FLUTTER → MECHANISMS → PULMONARY VEIN ISOLATION). In recent years, the attention of AF-related complications has promoted research on stroke and anticoagulation (ATRIOVENTRICULAR JUNCTION → SINUS RHYTHM → STROKE WARFARIN). We have witnessed the glorious era of maze procedure. With the development of minimally invasive technology, even modified maze procedure had to withdraw from the center of the stage and was replaced by pulmonary vein isolation (OPEN HEART SURGERY → MAZE PROCEDURE → SURGICAL ABLATION → COX MAZE PROCEDURE).

### Co-citation: journals, references, and authors

**Figure 5:**
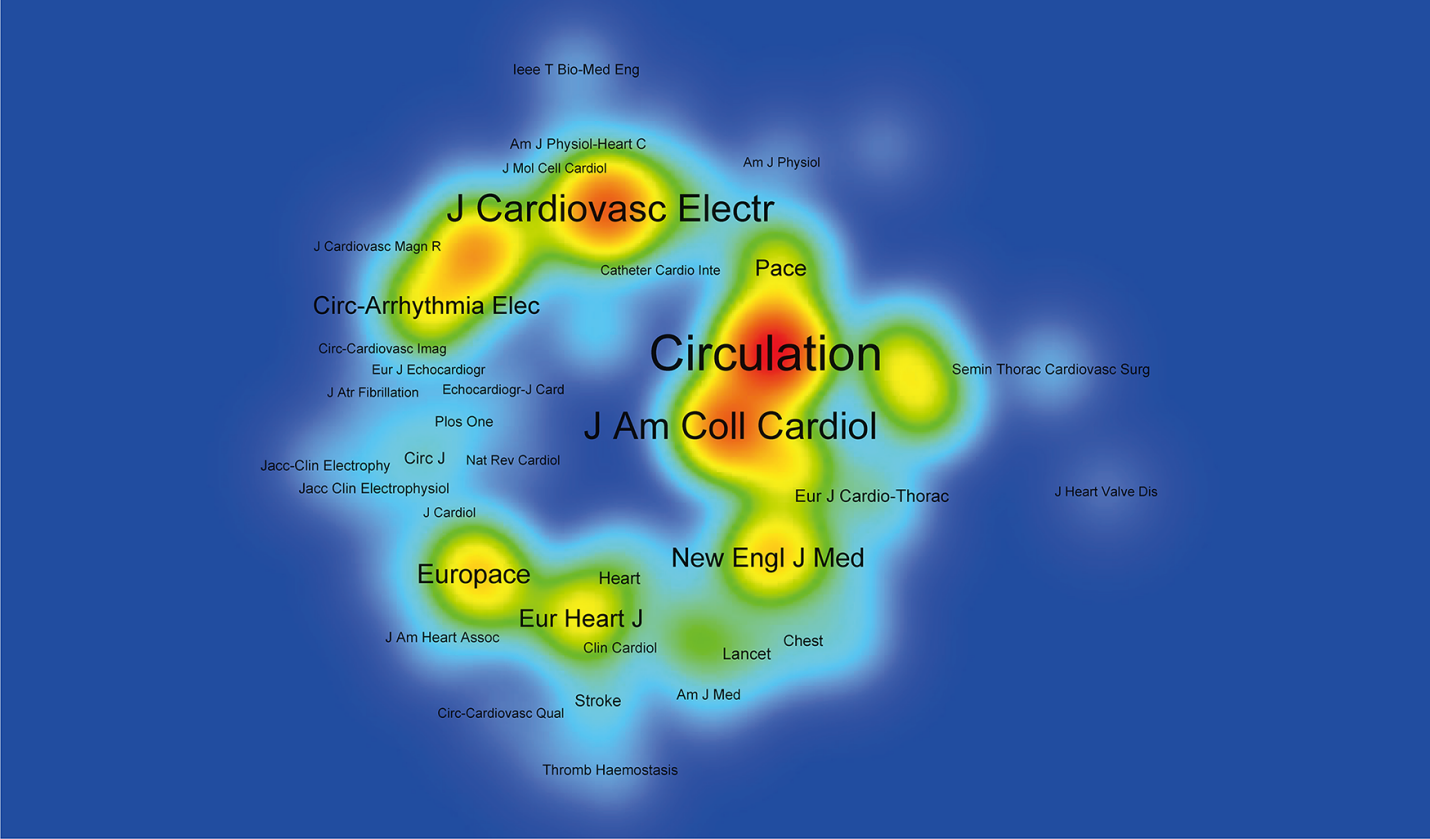
Density map of co-cited journals for the catheter ablation and surgical treatment of atrial fibrillation.

**Figure 6:**
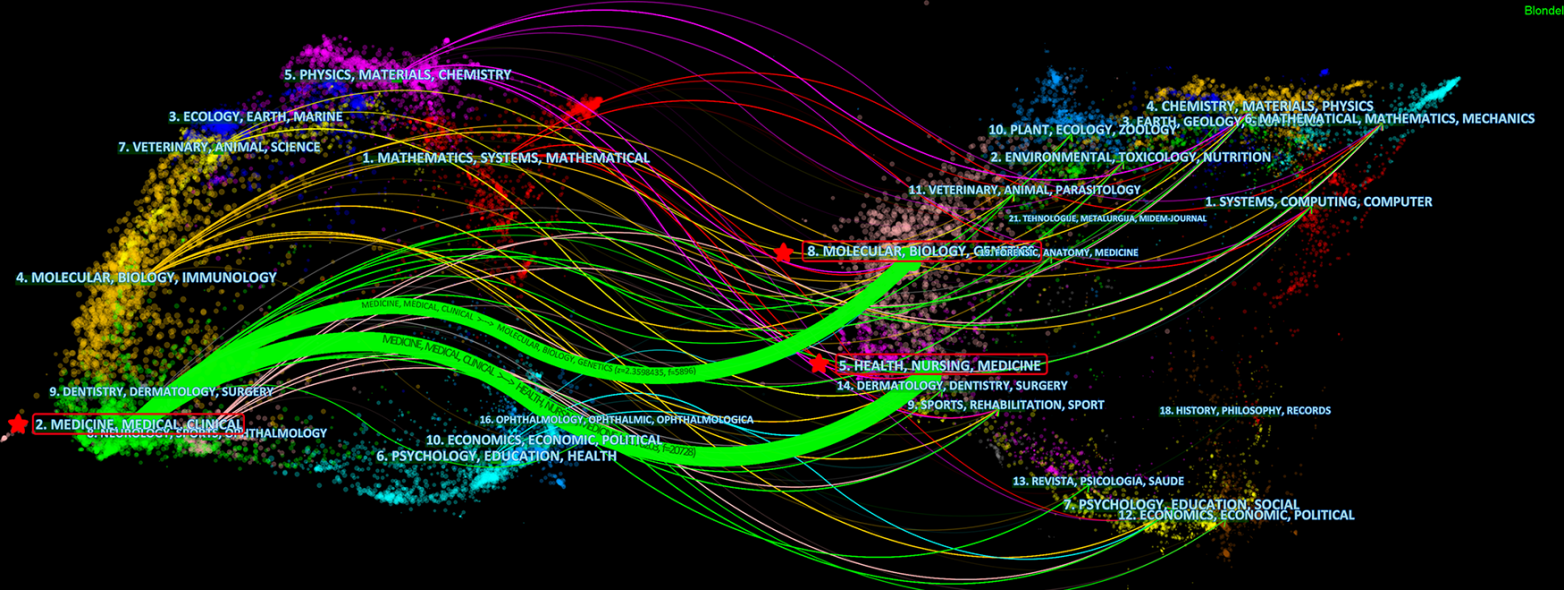
Dual-map overlay of journals for the catheter ablation and surgical treatment of atrial fibrillation.

Table 2 listed the top 10 co-cited journals, among which *Circulation* was cited for 39,911 times, ranking first. Followed by *Journal of Cardiovascular Electrophysiology* (Citations = 24,778) and *Journal of The American College of Cardiology* (Citations = 24,484). Figure 5 showed the hot map of co-cited journals, which required journals to have at least 300 co-citations, showing the distribution of highly cited journals. Figure 6 showed the dual-map overlay of journals. On the left was a map of cited journals, and on the right was a map of co-cited journals. This label represented the subject covered by the journal (24). Colored curves represented reference paths, originating from the left reference map and pointing to the right reference map. Two major citation routes were shown on the map. The green route refered to documents published in medicine/medical/clinical mostly cited journals in molecular/biology/genetics, and health/nursing/medicine.

**Table 2:** Top 10 co-cited journals for the catheter ablation and surgical treatment of atrial fibrillation.

Rank	Journal	IF	Citations
1	Circulation	29.690	39,911
2	Journal of Cardiovascular Electrophysiology	2.424	24,778
3	Journal of The American College of Cardiology	24.094	24,484
4	Heart Rhythm	6.343	17,010
5	Europace	5.214	11,639
6	New England Journal of Medicine	91.245	10,801
7	Circulation-Arrhythmia and Electrophysiology	6.568	9,782
8	American Journal of Cardiology	2.778	9,570
9	European Heart Journal	29.983	9,334
10	Pace-pacing and Clinical Electrophysiology	1.976	7,835

A total of 70,682 cited references were included in the analysis, and the top 10 co-cited references were listed in Table 3. Among them, there were two documents with more than 1,000 co-citation times. The first one was published on *New England Journal of Medicine* by Haissaguerre M et al. in 1998 (25), and the co-citation times were as high as 1991, occupying the absolute advantage. Secondly, two documents were cited 600–1000 times. There were four papers have been cited 500–600 times, respectively. References with Citation burst refers to documents that have been frequently cited over a period of time. The citation duration was set to the minimum of six, and a total of 31 co-cited literatures were included. As shown in Supplementary Appendix A Figure A2, the time interval was represented by the blue line, and the time interval for subjects to burst was represented by the red line. The red line represented the duration of the burst, and both ends of the red line represents the start and end years of the burst. Initially, there were three cited literatures starting from 1991, and all of them continued until 1996. There were two co-citations that lasted until 2021. The one with the highest intensity was written by Oral H in 2001 (26).

**Table 3:** Top 10 co-cited references for the catheter ablation and surgical treatment of atrial fibrillation.

Rank	Co-Cited reference	Citations
1	Haissaguerre M, Jais P, Shah DC, et al. Spontaneous initiation of atrial fibrillation by ectopic beats originating in the pulmonary veins. *N Engl J Med* 1998; 339 (10): 659–66.	1,991
2	Calkins H, Kuck KH, Cappato R, et al. 2012 HRS/EHRA/ECAS Expert Consensus Statement on Catheter and Surgical Ablation of atrial fibrillation: Recommendations for Patient Selection, Procedural Techniques, Patient Management and Follow-up, Definitions, Endpoints, and Research Trial Design. *Europace* 2012; 14 (4): 528–606. / *Heart Rhythm* 2012; 9 (4): 632–96	1,101
3	Nademanee K, McKenzie J, Kosar E, et al. A new approach for catheter ablation of atrial fibrillation: Mapping of the electrophysiologic substrate. *J Am Coll Cardiol* 2004; 43 (11): 2044–53.	746
4	Cappato R, Calkins H, Chen S-A, et al. Updated Worldwide Survey on the Methods, Efficacy, and Safety of Catheter Ablation for Human atrial fibrillation. *Circ-Arrhythmia Electrophysiol* 2010; 3 (1): 32–8.	644
5	Cappato R, Calkins H, Chen SA, et al. Worldwide survey on the methods, efficacy, and safety of catheter ablation for human atrial fibrillation. *Circulation* 2005; 111 (9): 1100–5.	534
6	Chen SA, Hsieh MH, Tai CT, et al. Initiation of atrial fibrillation by ectopic beats originating from the pulmonary veins—Electrophysiological characteristics, pharmacological responses, and effects of radiofrequency ablation. *Circulation* 1999; 100 (18): 1879–86.	533
7	Pappone C, Rosanio S, Oreto G, et al. Circumferential radiofrequency ablation of pulmonary vein ostia—A new anatomic approach for curing atrial fibrillation. *Circulation* 2000; 102 (21): 2619–28.	519
8	Calkins H, Brugada J, Packer DL, et al. HRS/EHRA/ECAS expert consensus statement on catheter and surgical ablation of atrial fibrillation: Recommendations for personnel, policy, procedures and follow-up. *Heart Rhythm* 2007; 4 (6): 816–61.	517
9	Cox JL, Schuessler RB, Dagostino HJ, et al. The Surgical-Treatment of Atrial-Fibrillation.3. Development of A Definitive Surgical-Procedure *J Thorac Cardiovasc Surg* 1991; 101 (4): 569–83.	484
10	Verma A, Jiang C-Y, Betts TR, et al. Approaches to Catheter Ablation for Persistent atrial fibrillation. *N Engl J Med* 2015; 372 (19): 1812–22.	477

Co-cited authors refer to the authors of the cited documents in the analyzed papers. A total of 36,839 authors were cited, and the top 10 co-cited authors were listed in Table 4. Among them, Haissaguerre M from Hopital Cardiologique du Haut-Leveque(France), was cited 5,039 times, occupying the first place with an absolute advantage. It was followed by Calkins H (Citations = 3,351) from Johns Hopkins University, and Cox JL (Citations = 3,020) from the University of Washington.

**Table 4:** Top 10 co-cited authors for the catheter ablation and surgical treatment of atrial fibrillation.

Rank	Co-Cited author	Institution	Citations
1	Haissaguerre M	Hopital Cardiologique du Haut-Leveque (France)	5,039
2	Calkins H	Johns Hopkins University (USA)	3,351
3	Cox, JL	University of Washington (USA)	3,020
4	Pappone, C	San Raffaele University Hospital (Italy)	2,751
5	Oral, H	Medical Center Dr, Ann Arbor, MI (USA)	2,488
6	Cappato, R	Milanese,Arrhythmia & Electrophysiol Center (Italy)	1,725
7	Verma, A	Montreal Heart Institute (Canada)	1,692
8	Jais, P	Hopital Cardiologique du Haut-Leveque (France)	1,676
9	Kirchhof, P	University Hospital Munster (Germany)	1,133
10	Di Biase, L	St Davis Medical Center (USA)	1,122

### Analysis of journals, countries, institutions, and authors

The 11,437 documents selected for inclusion were published in 719 journals. Among them, *Journal of Cardiovascular Electrophysiology* published the most papers (*N* = 1450, 11.38%), followed by *Europace* (*N* = 821, 6.45%), and *Journal of Interventional Cardiac Electrophysiology* (744, 5.84%). A total of 6,631 institutions from 90 countries participated in the study, with USA leading the way with 3,789 publications, accounting for 33.13%. The most productive institution was Mayo Clinic in the USA, accounting for 2.10%, ranking first. The most influential author was Natale A from St Davis Medical Center in USA, who has published 301 papers, accounting for 2.36%, and was cited 17,897 times. Followed by Jais P (*N* = 202, 1.59%, Citations = 21,855) and Haissaguerre M (*N* = 201, 1.58%, Citations = 20,598) from Hopital Cardiologique du Haut-Leveque. See Supplementary Appendix B for detailed summary information and cluster analysis of journals and co-authorship (countries, institutions, and authors).

## Discussion

With the development of the times and the progress of medical and health undertakings, the common AF has been focused on more and more people. Therefore, the research for the catheter ablation and surgical treatment of AF has been continuously deepened, and various new theories and technologies have been put forward constantly. This study is the first to provide a global, no time-limited bibliometric analysis of the catheter ablation and surgical treatment of AF's publication trends, journals, co-cited citations, co-cited authorship and keywords, and the relevant literatures in this research field were analyzed and revealed in depth. Cluster analysis and hotspot analysis reveal the research status and future research frontiers in this field. This analysis allows us to assess global output in this field, and make academic rankings, which can help fund supporters and policy makers to prioritize and allocate limited resources to authors and institutions that continue to produce high-quality work.

From the analysis of annual publications and annual relationship curves of the catheter ablation and surgical treatment of AF, we found that the first relevant literature appeared in 1985 (22) and there was a lack of published literature before 1990. After 1990, the annual growth rate of literature published in this field was positive, indicating that the research in this field was gradually paid more attention. The study of AF started early, with initial studies in the 1920s, but non-drug treatment studies started late and did not go far enough. Since the 1980s, there have been left atrial isolation, His bundle dissection, corridor surgery, atrial transection, and other surgical procedures. However, due to the great limitations of these surgical procedures, they cannot be effectively promoted (10, 27). Until 1987, the Cox-Maze Procedure performed by Cox JL became a milestone breakthrough in the development of the catheter ablation and surgical treatment of AF due to its good prognosis (11). And after being refined by Cox JL and his team, the third generation of Cox-Maze Procedure was introduced in 1992 and became the gold standard for the catheter ablation and surgical treatment of AF (12). Therefore, after 1990, AF treatment research has developed significantly, with more than 100 documents published in 1999. In recent years, with the continuous development of techniques to create scars without cutting tissue, people have reconsidered the maze surgery, and the use of new ablative energy has promoted the development of the fourth generation of Cox-Maze Procedure (13). As a result, there were more research directions to choose from. With 292 documents in 2005, 530 documents in 2011, 887 documents in 2020, and about 930 documents predicted in 2021, the research for the catheter ablation and surgical treatment of AF is entering an explosion. It can be foreseen that the accumulative amount of literature in this field will increase continuously in a certain period in the future.

The keywords check in this study showed that a total of 13,106 keywords were extracted from 1980 to 2021. In the frequency study of keywords, the total frequency of keywords was 118,843 times, 7,843 keywords (59.84%) appeared only once, 2,254 keywords (17.20%) appeared no less than five times, 1,206 keywords (9.20%) appeared no less than 10 times, 159 keywords (1.21%) appeared more than 100 times, indicating that the higher the frequency, the lower the number of keywords. Among the top 20 keywords with occurrence frequency (as shown in Table 1), in addition to some methodological keywords such as efficacy, management, follow-up, risk, etc., keywords such as atrial fibrillation, catheter ablation, radiofrequency ablation, and pulmonary vein isolation represented research hotspots and trends.

The co-occurrence analysis of keywords helps researchers to explore the topic distribution and emerging research trends of specific disciplines. 69 keywords with occurrence frequency no less than 250 times were clustered to form four categories. Cluster I with red color contained 26 keywords, mainly related to the epidemiology, treatment, prognosis, complications, and some methods of AF. AF is the most common arrhythmia to diagnose. As a major public medical challenge, AF occurs mainly in developed countries, but also in low-income and middle-income countries. About the prevalence of AF in 2017, there were 37.57 million prevalent cases and 3.05 million incident cases of AF globally, contributing to 287,241 deaths (28). In terms of mortality from AF, a landmark publication from the Framingham Heart Study showed that mortality rates were 50%–90% higher in participants with AF compared to those without AF (29). New research showed that the death rate from AF has decreased (30, 31). These improvements could be due to the progression of prevention of thromboembolic complications of AF (32, 33). The treatment of AF is a huge challenge as the incidence of AF continues to increase in an aging society. Adequate AF management requires implementation and adherence to evidence-based guidelines in clinical practice (33). However, management outcomes can be evaluated to determine the effectiveness of management programs and to improve atrial fibrillation care through other measures (34). Clinical indicators of AF as a measure of the severity and prognosis of patients, to guide clinicians to choose treatment. Short-term and long-term follow-up of patients with AF was important to assess the extent to which patients have recovered or deteriorated after treatment, and to make timely treatment adjustments. AF has a wide range of clinical risk factors, such as age, heart failure (HF), chronic obstructive pulmonary disease, and obesity. Meanwhile, risk factors and markers of underlying atrial cardiomyopathy severity (left atrial size, initial duration/burden of subclinical AF) were independent predictors of AF progression (35). Cluster II with green color contained 22 keywords, which were mainly related to the mechanism and the manifestations of AF. The occurrence of AF was influenced by many factors, involving the intersections of signaling pathways, biomacromolecules, and neurohumoral networks. From the perspective of electrophysiology, the ectopic activation of pulmonary veins started AF, the multi-wavelet return theory was used to explain the maintenance of AF, and several different mapping techniques of AF were used to map AF to further clarify the potential mechanism (36). At the same time, atrial tachycardia reconstruction was related to the underlying mechanisms of AF, because the initiation of AF by any mechanism may induce tachycardia reconstruction, thereby promoting the maintenance of AF through multi-circuit reentry (37). Atrial fibrosis also played a key role in the occurrence and persistence of AF. Increased deposition of extracellular matrix led to abnormal conduction through the atria, which formed the matrix for AF. Molecular pathways including renin-angiotensin system and TGF- B1 were involved in atrial fibrosis and participated in the occurrence and development of AF through structural remodeling (38). Both electrophysiological remodeling and tissue remodeling promoted AF, and the former was highly reversible, while the latter changed slowly but was more difficult to reverse. Abnormal Ca^2+^ regulation also contributed to AF. Intracellular Ca^2+^ imbalance caused intracellular Ca^2+^ overload and triggered arrhythmia (39). The study on the mechanism of AF provides new ideas for the treatment and equipment of AF. Cluster III with blue color contained 16 keywords, mainly related to the newly developed ablative techniques for AF, as well as some methodological terms. Focal discharge from the pulmonary veins was widely believed to be the cause of AF (40). Over the past 20 years, pulmonary vein isolation has advanced significantly and has become the cornerstone of catheter-based AF therapy. Since the development of the fourth generation of Cox-Maze Procedure in 2004, ablation using new energy has replaced the traditional complex “cutting and sutured” technology (13). After more than a decade of development, it has become more mature and become the mainstream of AF treatment. The commonly used radiofrequency ablation with catheter uses heat energy to heat tissues and permanently damage the tissue structure and function of cells to achieve the purpose of ablation. There are two types of radiofrequency ablation: unipolar radiofrequency ablation and bipolar radiofrequency ablation, each of which has its own advantages and disadvantages. The former covers a wide range, but has low efficiency and is easy to form esophageal fistula. The latter has higher safety, shorter operation time and higher success rate. In addition, cryoballoon ablation is commonly used clinically. There are other energy ablation technologies, such as microwave ablation, high intensity focused ultrasound ablation, laser ablation, etc. Different ablative methods are used for different types of AF, and the ablative technology is constantly improved, and the development in recent years has been closer to minimally invasive and interventional (41). Although AF ablation has many advantages, the recurrence of AF and the occurrence of serious clinical complications indicate that AF ablation also has a risk of failure. Risk factors for recurrence are dependent on the type and mechanism of AF. Persistent AF has a high rate of recurrence (42) and exhibits a high rate of recurrence of one or more of three mechanisms: automatic foci producing spontaneous discharges (43), randomly propagating wavelets (44), or discrete areas of localized reentry (45). Female sex and increased left atrial diameter were also significantly associated with recurrence (46). Cluster IV consists of only five keywords, which was mainly related to the traditional surgical treatment of AF. The introduction of Cox-Maze Procedure in 1987 was a breakthrough in the catheter ablation and surgical treatment of AF and the cornerstone of subsequent catheter ablation and surgical treatment (11). After continuous improvement, the third generation of Cox-Maze Procedure has become the gold standard (12). Even today, although there are more simple and convenient surgical methods, its good prognosis is still irreplaceable. Of note is the comparison between percutaneous catheter ablation and surgical treatment. The most recent guidelines on AF management from Canadian Cardiovascular Society/Canadian Heart Rhythm Society (47) and American College of Cardiology (ACC)/American Heart Association (AHA) (48) do not give clear recommendations. The latest guidelines of European Society of Cardiology (ESC) suggest that compared with percutaneous catheter ablation, surgical treatment has a higher incidence of complications and a longer hospital stay, but a significantly lower rate of repeat ablation and recurrence, which seems to be a more reasonable choice for patients with previous failed or high risk of failure of catheter ablation (49).

Keyword co-occurrence analysis can reflect hot topics in research, and keyword bursts may indicate frontier topics or emerging trends (50). From the burst chart, we made a horizontal and vertical comparative analysis from the two dimensions of time start and end and burst strength, and were particularly interested in the key words of research significance that have begun to burst in recent years. There were 12 keywords that would continue through 2021. According to the analysis, 2nd generation cryoballoon, atrial fibrosis, substrate modification, and atrial fibrillation anticoagulation seem to be research trends in recent years. Cryoablation had been proposed earlier, but the first generation of ablation had the problems of long time and low efficiency, and has not obvious advantages compared with radiofrequencies ablation (51). However, with the second generation of cryoablation proposed, the surgical time and complications were reduced, and the permanent of pulmonary veins isolation was achieved with greater efficiency. The traditional radiofrequency ablation is expected to be replaced by cryoablation (51, 52). Atrial fibrosis can carry out structural remodeling of the atria and lead to conduction disorders to maintain AF. Inflammation can activate fibroblasts and lead to atrial fibrosis (53). Clinical intervention in early inflammation may be an effective measure for the treatment of AF (54). Conventional ablation protocols use progressive ablation guided by the termination of arrhythmia, and pulmonary vein isolation is the cornerstone. However, substrate modification apart from pulmonary vein isolation, which has been proposed in recent years, is a potential research site with shorter surgical duration, less fluoroscopy, and less damage to normal tissues (55, 56). Some hot spots found by burst keywords will be the hot frontier of future research. These hot issues should have higher priority and clinical value in future research.

Co-citation means that the literatures of two authors/journals are cited by the third author at the same time, and the two authors/journals/references are co-cited (57). Co-citation intensity is shown as the thickness of the links between nodes in the cluster network map. Of the top 10 co-cited journals listed, there were some top journals, such as *Circulation*, *Journal of The American College of Cardiology*, *New England Journal of Medicine*, and *European Heart Journal*. There were cardiovascular journals such as *Heart Rhythm*, *Europace*, *Circulation-Right Atriummia*, *and Electrophysiology*. Literature citation in co-cited journals still followed Bradford's law, resulting in a large proportion of citations coming from a few core journals. The average IF of the top 10 co-cited journals was 20.032, and seven of them had an IF above 5.000, indicating that journals with high IF were more likely to be co-cited. Most of the top ten co-cited literatures were reviews, summarizing some classical theories and basic techniques in this field. The strongest burst appeared in three papers (26, 58, 59), with high citation duration of six years. These three cited papers with high citation duration represented major breakthroughs in studies for the catheter ablation and surgical treatment of AF at different stages and were of great significance. These three documents separately identified the end point of partial catheter ablation for pulmonary vein lesions causing AF (58), the clinical efficacy of segmental pulmonary vein isolation in patients with AF in different types of AF (26), and the advantages of circum-pulmonary vein isolation during complete vagal denervation (59). The start and end times of cited references were gradually replaced, which represented the development trend in this field. Among the top 10 co-cited authors, Haissaguerre M ranked first, while he ranked third in the number of publications. It showed that Haissaguerre M was a real leader in this field, and his research played a cornerstone role in the field for the catheter ablation and surgical treatment of AF. Jais P, Calkins H, and Di Biase L are in a leading position in both the number of documents published and the number of co-citations, which fully demonstrated the great contribution and international influence of them in the field for the catheter ablation and surgical treatment of AF.

There are still some limitations. (1) Only the relevant literatures included in WoSCC were analyzed, including only three types of literatures (article, review, and early access), almost all of which were in English. As a result, there may be partial bias in the included literature sources. (2) In literature screening, although three researchers were involved, there was still bias due to personal factors such as knowledge background and opinions. (3) Before the analysis, we manually standardized and corrected some results including keywords to reduce the deviation caused by different expressions of the same concept, but it could not be eliminated completely.

## Conclusion

From the perspectives of visualization and bibliometrics, this study used three bibliometrics software to analyze the literature related to the catheter ablation and surgical treatment of AF from 1980 to 2021, including publication trend, country, institution, author, journal, keywords, and co-citation references. We analyzed from three aspects of frequency, clustering, and hot spots, so that predicted the future research frontiers. In the research trend, the annual publication rate gradually increased. Developed countries led by the USA were the leaders in the field for the catheter ablation and surgical treatment of AF, while China has made great progress in recent years and ranked third in the number of documents published. Currently, atrial fibrosis, substrate modification, minimally invasive and access surgery will become the research focus and frontier in the next few years.

## Data Availability

The original contributions presented in the study are included in the article/**Supplementary Material**, further inquiries can be directed to the corresponding author/s.
