# Corrigendum: Global research trends in catheter ablation and surgical treatment of atrial fibrillation: A bibliometric analysis and science mapping

**DOI:** 10.3389/fsurg.2023.1155709

**Published:** 2023-02-22

**Authors:** Xiang Gao, Kai Liu, Xinke Zhao, Xinfang Lv, Xue Wu, Chunzhen Ren, Qilin Chen, Yingdong Li

**Affiliations:** ^1^ Gansu University of Chinese Medicine, Lanzhou, China; ^2^ Affiliated Hospital of Gansu University of Chinese Medicine, Lanzhou, China

**Keywords:** atrial fibrillation, catheter ablation, surgical treatment, bibliometric analysis, science mapping analysis, citation analysis

A Corrigendum on Global research trends in catheter ablation and surgical treatment of atrial fibrillation: A bibliometric analysis and science mapping By Gao X, Liu K, Zhao X, Lv X, Wu X, Ren C, Chen Q and Li Y. (2023) Front. Surg. 9:1048454. doi: 10.3389/fsurg.2022.1048454

**Incorrect Affiliation**

In the published article, there was an error in affiliation(s) [1, 2]. Instead of “[1 Department of Cardiovascular Surgery, Gansu Provincial Hospital, Gansu University of Chinese Medicine, Lanzhou, China. 2 Department of Cardiovascular Surgery, Gansu Provincial Hospital, Affiliated Hospital of Gansu University of Chinese Medicine, Lanzhou, China.]”, it should be “[1 Gansu University of Chinese Medicine, Lanzhou, China. 2 Affiliated Hospital of Gansu University of Chinese Medicine, Lanzhou, China.]”.

The authors apologize for this error and state that this does not change the scientific conclusions of the article in any way. The original article has been updated.

