# Supplementary material for: Global research trends in catheter ablation and surgical treatment of atrial fibrillation: A bibliometric analysis and science mapping
Source: Front Surg. 2023 Jan 6;9:1048454. doi: 10.3389/fsurg.2022.1048454 (PMC9852516; doi:10.3389/fsurg.2022.1048454)
Supplement: Supplementary file 1 [file Datasheet1.pdf]

Figure A1 Top 65 Keywords with the Strongest Citation Bursts

Keywords	Year	Strength	Begin	End	2010 - 2021
atrium	2010	11.22	**2010**	2013	
circumferential ablation	2010	8.87	**2010**	2013	
image integration	2010	8.78	**2010**	2013	
vein antrum isolation	2010	8.23	**2010**	2013	
sinus	2010	7.71	**2010**	2013	
repair	2010	6.57	**2010**	2014	
long term	2010	6.33	**2010**	2014	
cfae	2010	6.05	**2010**	2013	
complex fractionated atrial electrogram	2010	5.71	**2010**	2015	
isthmus	2010	5.62	**2010**	2014	
initial experience	2010	5.5	**2010**	2015	
angiography	2010	5.34	**2010**	2013	
parkinson white syndrome	2010	5.24	**2010**	2013	
term	2010	5.19	**2010**	2014	
double lasso technique	2010	5.01	**2010**	2014	
stenosis	2010	4.86	**2010**	2013	
perforation	2010	4.84	**2010**	2013	
posterior left atrium	2010	4.76	**2010**	2013	
dronedarone	2010	4.48	**2010**	2013	
remodeling	2010	4.41	**2010**	2015	
canine model	2010	4.37	**2010**	2013	
electrophysiological characteristics	2010	3.87	**2010**	2013	
refractoriness	2010	3.74	**2010**	2014	
congestive heart failure	2010	3.65	**2010**	2014	
integration	2010	3.6	**2010**	2013	
navigation system	2010	3.53	**2010**	2014	
ultrasound	2010	3.17	**2010**	2015	
chronic atrial fibrillation	2010	2.82	**2010**	2013	
anatomic approach	2010	2.8	**2010**	2014	
acute myocardial infarction	2010	2.6	**2010**	2016	
international normalized ratio	2010	7.63	**2011**	2014	
imaging	2010	6.11	**2011**	2014	
left ventricular dysfunction	2010	4.3	**2011**	2014	
sinusrhythm	2010	4.09	**2011**	2014	
cryoballoon technique	2010	3.35	**2011**	2014	
fractionation	2010	2.72	**2011**	2015	
fractionated electrogram	2010	2.67	**2011**	2017	
task force	2010	14.61	**2012**	2015	
navigation	2010	4.61	**2012**	2015	
lone atrial fibrillation	2010	2.98	**2012**	2016	
circuit	2010	3.11	**2013**	2017	
heart rhythm association	2010	10.14	**2014**	2017	
periprocedural anticoagulation	2010	4.51	**2014**	2018	
radiofrequency catheter	2010	3.76	**2014**	2017	
electrocardiogram	2010	3.76	**2014**	2017	
tissue	2010	5.74	**2015**	2018	
burden	2010	3.5	**2015**	2021	
complicating ablation	2010	2.74	**2015**	2018	
2nd generation cryoballoon	2010	13.5	**2016**	2021	
rotor modulation	2010	7.33	**2016**	2019	
phrenic nerve injury	2010	6.98	**2016**	2019	
focal impulse	2010	6.3	**2016**	2019	
magnetic resonance	2010	5.45	**2016**	2019	
freeze	2010	5.25	**2016**	2019	
long term outcm	2010	5.16	**2016**	2021	
apixaban	2010	5.07	**2016**	2021	
cardiac magnetic resonance	2010	4.34	**2016**	2019	
substrate modification	2010	2.59	**2016**	2021	
european association	2010	6	**2017**	2021	
2nd generationcryoballoon	2010	5.7	**2017**	2021	
heart rate variability	2010	5.01	**2017**	2021	
collaboration	2010	4.02	**2017**	2021	
dynamics	2010	3.76	**2017**	2021	
atrial fibrosis	2010	3.06	**2017**	2021	
expert consensus	2010	2.99	**2017**	2021	

Figure A2 Top 31 References with the Strongest Citation Bursts

References	Year	Strength	Begin	End	1985 - 2021
COX JL, 1991, J THORAC CARDIOV SUR, V101, P569, DOI 10.1016/s0022-5223(19)36684-x, DOI	1991	25.32	**1991**	1996	
COX JL, 1991, J THORAC CARDIOV SUR, V101, P406, DOI 10.1016/s0022-5223(19)36723-6, DOI	1991	17.98	**1991**	1996	
CALKINS H, 1991, NEW ENGL J MED, V324, P1612, DOI 10.1056/NEJM199106063242302, DOI	1991	11.98	**1991**	1996	
COX JL, 1995, J THORAC CARDIOV SUR, V110, P473, DOI 10.1016/S0022-5223(95)70244-X, DOI	1995	29.22	**1995**	2000	
Prystowsky EN, 1996, CIRCULATION, V93, P1262, DOI 10.1161/01.CIR.93.6.1262, DOI	1996	15.32	**1996**	2001	
Cauchemez B, 1996, CIRCULATION, V93, P284, DOI 10.1161/01.CIR.93.2.284, DOI	1996	15.32	**1996**	2001	
Gaita F, 1998, CIRCULATION, V97, P2136, DOI 10.1161/01.CIR.97.21.2136, DOI	1998	39.45	**1998**	2003	
Kottkamp H, 1999, J CARDIOVASC ELECTR, V10, P772, DOI 10.1111/j.1540-8167.1999.tb00256.x, DOI	1999	23.58	**1999**	2004	
Nitta T, 1999, ANN THORAC SURG, V67, P27, DOI 10.1016/S0003-4975(98)01364-2, DOI	1999	13.47	**1999**	2004	
Haissaguerre M, 2000, CIRCULATION, V101, P1409, DOI 10.1161/01.CIR.101.12.1409, DOI	2000	75.46	**2000**	2005	
Haissaguerre M, 2000, J CARDIOVASC ELECTR, V11, P2, DOI 10.1111/j.1540-8167.2000.tb00727.x, DOI	2000	20.41	**2000**	2005	
Oral H, 2002, CIRCULATION, V105, P1077, DOI 10.1161/hc0902.104712, DOI	2002	86.78	**2002**	2007	
Nattel S, 2002, NATURE, V415, P219, DOI 10.1038/415219a, DOI	2002	7.76	**2002**	2007	
Doll N, 2003, J THORAC CARDIOV SUR, V125, P836, DOI 10.1067/mtc.2003.165, DOI	2003	28.41	**2003**	2008	
Pappone C, 2004, CIRCULATION, V109, P327, DOI 10.1161/01.CIR.0000112641.16340.C7, DOI	2004	57.33	**2004**	2009	
Chen MS, 2004, J AM COLL CARDIOL, V43, P1004, DOI 10.1016/j.jacc.2003.09.056, DOI	2004	12.98	**2004**	2009	
Ouyang FF, 2005, CIRCULATION, V111, P127, DOI 10.1161/01.CIR.0000151289.73085.36, DOI	2005	36.23	**2005**	2010	
Gaynor SL, 2005, J THORAC CARDIOV SUR, V129, P104, DOI 10.1016/j.jtcvs.2004.08.042, DOI	2005	19.35	**2005**	2010	
Gaita F, 2005, CIRCULATION, V111, P136, DOI 10.1161/01.CIR.0000151310.00337.FA, DOI	2005	18.51	**2005**	2010	
Chugh A, 2005, HEART RHYTHM, V2, P464, DOI 10.1016/j.hrthm.2005.01.027, DOI	2005	15.14	**2005**	2010	
Pappone C, 2006, J AM COLL CARDIOL, V47, P1390, DOI 10.1016/j.jacc.2005.11.058, DOI	2006	18.43	**2006**	2011	
Takahashi Y, 2008, J AM COLL CARDIOL, V51, P1003, DOI 10.1016/j.jacc.2007.10.056, DOI	2008	19.65	**2008**	2013	
Chun KRJ, 2009, EUR HEART J, V30, P699, DOI 10.1093/eurheartj/ehn570, DOI	2009	25.67	**2009**	2014	
Boersma LVA, 2012, CIRCULATION, V125, P23, DOI 10.1161/CIRCULATIONAHA.111.074047, DOI	2012	26.63	**2012**	2017	
Kuck KH, 2012, HEART RHYTHM, V9, P18, DOI 10.1016/j.hrthm.2011.08.021, DOI	2012	23.34	**2012**	2017	
Shah RU, 2012, J AM COLL CARDIOL, V59, P143, DOI 10.1016/j.jacc.2011.08.068, DOI	2012	17.86	**2012**	2017	
Pison L, 2012, J AM COLL CARDIOL, V60, P54, DOI 10.1016/j.jacc.2011.12.055, DOI	2012	17.49	**2012**	2017	
Morillo CA, 2014, JAMA-J AM MED ASSOC, V311, P692, DOI 10.1001/jama.2014.467, DOI	2014	31.71	**2014**	2019	
Narayan SM, 2014, J AM COLL CARDIOL, V63, P1761, DOI 10.1016/j.jacc.2014.02.543, DOI	2014	24.77	**2014**	2019	
Scherr D, 2015, CIRC-ARRHYTHMIA ELEC, V8, P18, DOI 10.1161/CIRCEP.114.001943, DOI	2015	29.14	**2015**	2021	
Ciconte G, 2015, HEART RHYTHM, V12, P673, DOI 10.1016/j.hrthm.2014.12.026, DOI	2015	15.3	**2015**	2021	
