# Supplementary material for: Global research trends in catheter ablation and surgical treatment of atrial fibrillation: A bibliometric analysis and science mapping
Source: Front Surg. 2023 Jan 6;9:1048454. doi: 10.3389/fsurg.2022.1048454 (PMC9852516; doi:10.3389/fsurg.2022.1048454)
Supplement: Supplementary file 2 [file Datasheet2.pdf]

Appendix B Detailed summary information and cluster analysis of journals and co-authorship (countries, institutions, and authors).

Analysis of journals

The 11,437 documents selected for inclusion were published in 719 journals. The top 20 most productive journals were listed in **Table B1**, with at least 85 papers published in each journal, and they published 7,051 papers (61.65%) of the total number of documents. Among them, *Journal of Cardiovascular Electrophysiology* published the most papers (N=1450, 11.38%), followed by *Europace* (N=821, 6.45%), *Journal of Interventional Cardiac Electrophysiology* (744, 5.84%) and *Heart Rhythm* (695, 5.46%). The average IF of the top 20 most productive journals was 7.746. In terms of journal origin, a large proportion of the top 20 journals were from the USA (11, 55%), followed by the Netherlands (3, 15%) and UK (3, 15%).

Table B1 Top 20 journals for the catheter ablation and surgical treatment of atrial fibrillation

Rank	Journals	N (%)	Cations	IF	Country
1	Journal of Cardiovascular Electrophysiology	1,450 (11.38%)	40,714	2.424	USA
2	Europace	821 (6.45%)	28,657	5.214	UK
3	Journal of Interventional Cardiac Electrophysiology	744 (5.84%)	8,599	1.900	Netherlands
4	Heart Rhythm	695 (5.46%)	28,707	6.343	USA
5	Pace-Pacing and Clinical Electrophysiology	646 (5.07%)	9,037	1.976	USA
6	Circulation-Arrhythmia and Electrophysiology	381 (2.99%)	15,999	6.568	USA
7	Circulation	270 (2.12%)	45,554	29.690	USA
8	International Journal of Cardiology	259 (2.03%)	3,499	4.164	Ireland
9	Annals of Thoracic Surgery	251 (1.97%)	8,863	4.330	USA
10	Journal of The American College of Cardiology	219 (1.72%)	29,692	24.094	USA
11	American Journal of Cardiology	201 (1.58%)	6,279	2.778	USA
12	Circulation Journal	191 (1.50%)	3,183	2.993	Japan
13	Journal of Thoracic and Cardiovascular Surgery	171 (1.34%)	9,300	5.209	USA
14	European Heart Journal	134 (1.05%)	19,767	29.983	UK
15	European Journal of Cardio-Thoracic Surgery	134 (1.05%)	4,404	4.191	Netherlands
16	Jacc-Clinical Electrophysiology	107 (0.84%)	1,133	6.375	USA
17	Heart and Vessels	103 (0.81%)	404	2.037	Japan
18	Journal of Cardiology	95 (0.75%)	794	3.159	Netherlands
19	Heart	91 (0.71%)	2,828	5.994	UK
20	Journal of The American Heart Association	88 (0.69%)	1,686	5.501	USA

Note: IF: Impact factor (InCites Journal Citation Reports dataset updated Jun 29, 2021), **JCR**: Journal Citation Reports, **SCIE**: Science Citation Index Expanded.

Journal analysis showed that 11,437 documents were published in 719 journals with an average publication volume of 15.91. In fact, 69.68% of the journals published no more than five documents, only 10.29% of the journals published more than 20 documents, and the number of papers published in the top 20 journals accounted for 61.65% of the total number of papers. Most of these journals were in the field of cardiology. It indicated that publication was distributed in accordance with Bradford's Law, which states that most papers on specialized topics are distributed by a limited number of specialized journals (journals on cardiac & cardiovascular systems). In terms of the number of published papers, *Journal of Cardiovascular Electrophysiology* (N=1,450), *Europace* (N=821) and *Journal of Interventional Cardiac Electrophysiology* (N=744) ranked the top three, yet in terms of the cited number, *Circulation* ranked the seventh in terms of the

number of published papers, ranking the first with an absolute advantage (Cited number =45,554). Although the cited number of *Journal of Cardiovascular Electrophysiology* (Cited number =40,714) and *Europace* (Cited number =28,657) was still high, it was achieved since their high number of papers, and the average number of citations was still far from that of *Circulation*. Combined with IF, it could be concluded that the average number of citations with high IF was also high, especially *Circulation* and *European Heart Journal*. The top 20 journals were all from developed countries, half of which were from USA, reflecting the huge advantages of developed countries in the field of the catheter ablation and surgical treatment of AF research.

Co-authorship: countries

All the publications (N =11,437) were attributable to 90 countries. The bar chart in **Figure B1** listed the countries with the most publications. Among them, the USA had the largest contribution, with 3,789 publications, accounting for 33.13%. Germany (N=1558, 13.62%), China (N=1328, 11.61%) and Japan (N=1250, 10.93%) followed suit and broke through 1,000 publications. It was followed by the UK (N=866, 7.57%), Italy (N=865, 7.56%) and France (N=619, 5.41%). **Figure B2** showed a visualization map of the collaboration between different countries with at least 10 publications. The visual map showed 47 countries in five different clusters, each with a different color, representing a different close collaboration. The USA had the strongest partner network, with a total link strength of 2,308, followed by Germany (Total link strength=1,836), UK (Total link strength=1,567) and Italy (Total link strength=1,465). Link strength was shown on the graph as the thickness of the lines between different nodes.

A total of 90 countries have participated in the research and published articles for the catheter ablation and surgical treatment of AF, suggesting that this research field has attracted the attention at the global level. In terms of the total number of publications, the USA was by far the most dominant, producing more than twice as much as the second most prolific country, Germany. This may be due to the historical advantages of USA in this field, including the medical field in general. Among the top 20 countries, there were only five developing countries, namely China, Poland, Denmark, Turkey, and Brazil, with a total publication volume of 1,980, accounting for 17.31%. However, excluding China, the other four countries had a total publication volume of 652, accounting for only 5.70%. There are several relevant factors for the huge difference in the number of publications in this field between developed and developing countries. First of all, developed countries have strong economic and industrial strength, can be self-sufficient in high-quality materials required for AF surgery, and the social welfare security mechanism is perfect. The historical advantages of developed countries in this field cannot be ignored, and the long-term and inheritable nature of the research content makes the research in developed countries more in-depth, extensive, and forward-looking. At the same time, the strong economic strength of developed countries has provided higher research and development funds for atrial fibrillation surgery research, which allows scholars to conduct research without being troubled by public relations and funding problems. The relatively free research environment is also an influence factor. Compared with developing countries, developed countries have a more perfect project evaluation and talent evaluation mechanism, which enables scholars to focus more on research and thus have more academic output. Therefore, developing countries should strengthen cooperation and exchanges with developed countries, learn advanced research methods and technologies, to promote the development of the research for the catheter ablation and surgical treatment of AF.

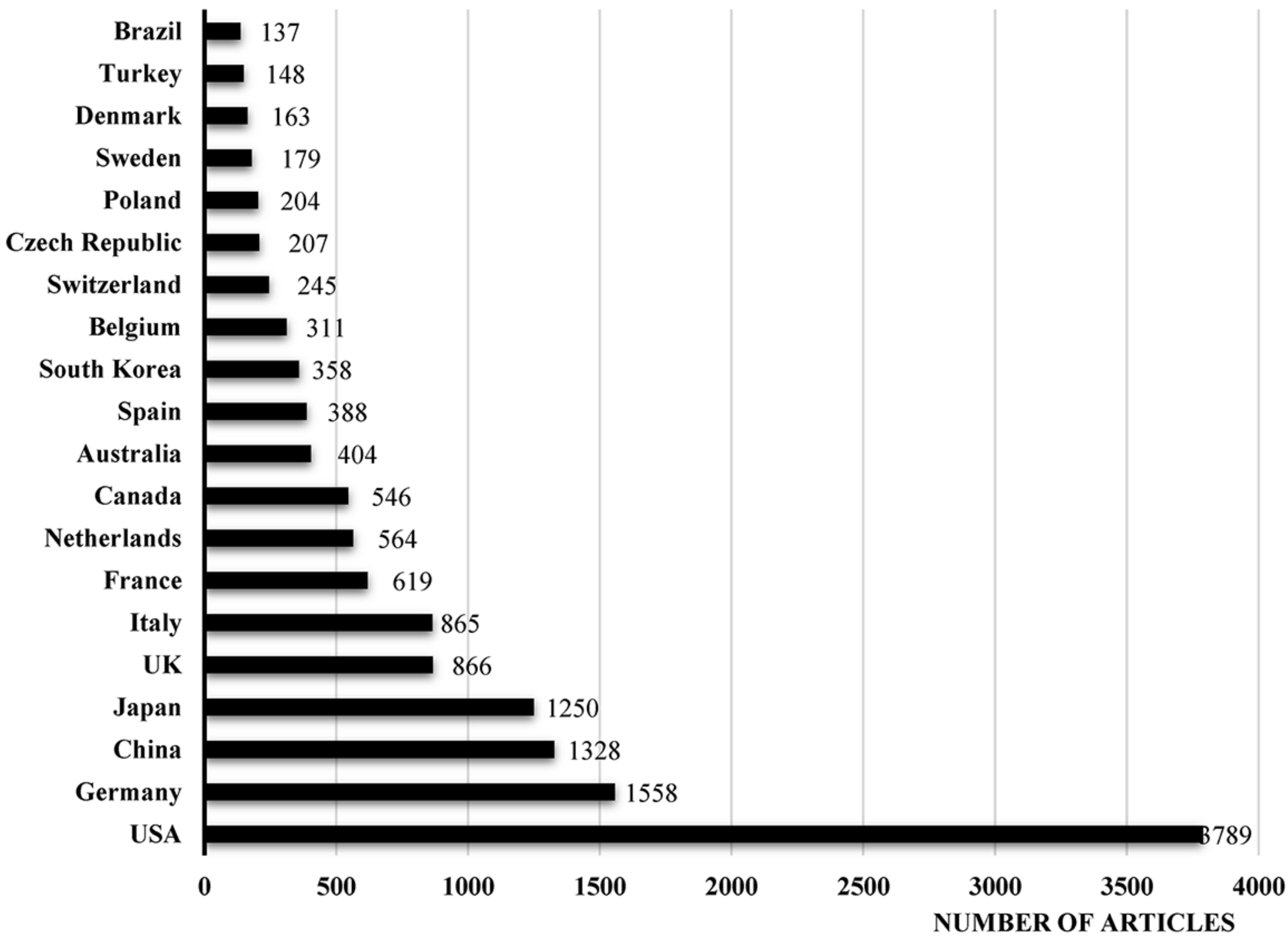

Figure B1 Top 20 countries with the most publications for the catheter ablation and surgical treatment of atrial fibrillation

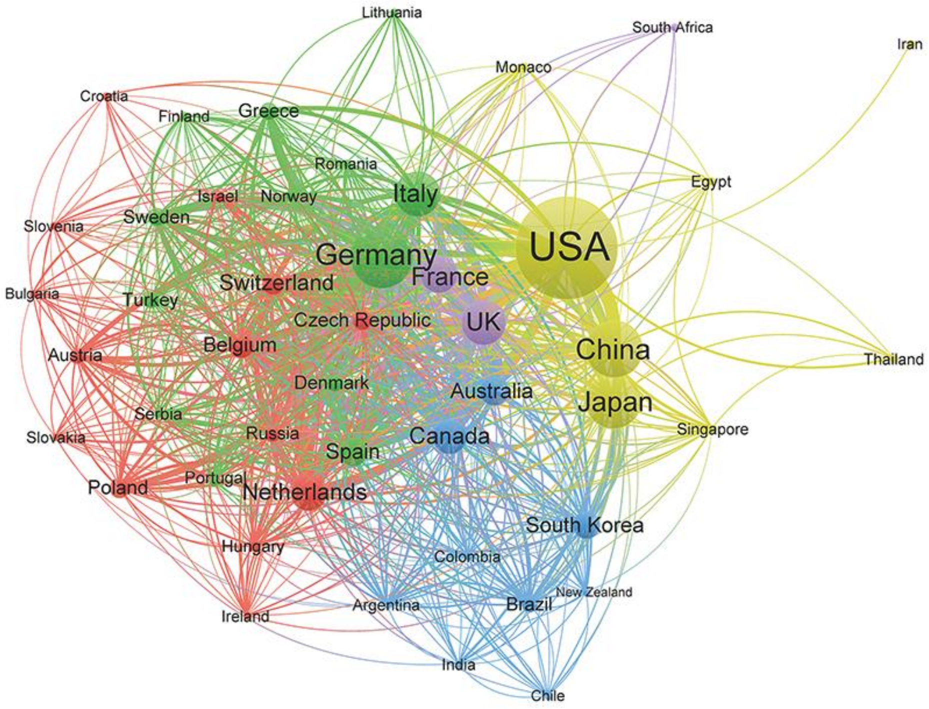

Figure B2 Network map of cooperation between countries for the catheter ablation and surgical treatment of atrial fibrillation

Co-authorship: institutions

A total of 6,631 institutions contributed to publications for the catheter ablation and surgical treatment of AF. Table B2 listed the top 20 institutions. Mayo Clinic in USA published 268 papers in this field, accounting for 2.10%, ranking first. In addition, Cleveland Clinic Foundation in USA published more than 200 papers (N=231, 1.81%), followed by National Yang-Ming University in Taiwan, China (N=191, 1.50%) and Capital Medical University in China (N=189, 1.48%). The other three published more than 180 papers were Harvard University in USA (N=188, 1.48%), Stanford University in USA (N=184, 1.44%) and St. David's Medical Center in USA (N=181, 1.42%). In terms of the origin of institutions, the USA had the largest share of institutions (10, 50%), followed by China (4, 20%), with Germany and Japan equally represented (2, 10%). As for average Citations, Hopital Cardiologique du Haut-Leveque from France ranked first (124.56), followed by Cleveland Clinic Foundation (79.61) and Massachusetts General Hospital (69.60) from USA. And University of Bordeaux (61.06) in France also surpassed 60. The visual network map in Figure B3 showed the cooperation between 45 institutions with at least 80 papers, which was divided into six clusters, respectively replaced by different colors. The more papers the institutions had, the closer the cooperation was, which was represented by large nodes and thick connecting lines.

Table B2 Top 20 institutions for the catheter ablation and surgical treatment of atrial fibrillation

Rank	Institution	N (%)	Citations	Citations /N
1	Mayo Clinic (USA)	268 (2.10%)	11,614	43.34
2	Cleveland Clinic Foundation (USA)	231 (1.81%)	18,391	79.61
3	National Yang-Ming University (Taiwan, China)	191 (1.50%)	7,173	37.55
4	Capital Medical University (China)	189 (1.48%)	2,147	11.36
5	Harvard University (USA)	188 (1.48%)	8,175	43.48
6	Stanford University (USA)	184 (1.44%)	6,622	35.99
7	St Davis Medical Center (USA)	181 (1.42%)	9,835	54.34
8	Leipzig University (Germany)	176 (1.38%)	7,156	40.66
9	The University of Michigan (USA)	163 (1.28%)	9,709	59.56
10	Asklepios Klinik St Georg (Germany)	163 (1.28%)	6,588	40.42
11	University of Washington (USA)	156 (1.22%)	8,887	56.97
12	Duke University (USA)	149 (1.17%)	4,760	31.95
13	Taipei Veterans General Hospital (Taiwan, China)	148 (1.16%)	4,187	28.29
14	Tokyo Medical and Dental University (Japan)	145 (1.14%)	2,048	14.12
15	Massachusetts General Hospital (USA)	144 (1.13%)	10,023	69.60
16	Shanghai Jiao Tong University (China)	142 (1.11%)	1,102	7.76
17	Johns Hopkins University (USA)	140 (1.10%)	8,292	59.23
18	Yonsei University (South Korea)	130 (1.02%)	1,674	12.88
19	University of Bordeaux (France)	130 (1.02%)	7,938	61.06
20	Hopital Cardiologique du Haut-Leveque (France)	126 (0.99%)	15,694	124.56

The top 20 most prolific institutions published 3,344 documents, accounting for 29.24% of the total number of published papers. Of these 20 institutions, seven are hospitals or medical centers and 13 are universities. The global distribution of institutions was consistent with the global distribution of countries, with the USA still leading the way with half of the institutions from USA, followed by China, indicating that China has also made great progress in research in this field. It was important to note that the ranking of average number of citations and the ranking of number of articles were not in one-to-one correspondence, such as the most obvious one was Hopital Cardiologique du Haut-Leveque in France. Although the number of documents published (N=126, 0.99%) ranked 20th, the number of articles cited (124.56) was as high as the first, indicating

that its articles had great influence and high quality. Cluster analysis showed that cooperation between countries and institutions was not affected by geographical location. The high link strength indicated that the collaboration between institutions was extensive, once again confirming that the treatment of AF was a globally recognized proposition and showing good prospects for research. Multiple clusters centered on the USA showed stronger linkage strength, suggesting that collaboration among these high-yielding countries and institutions further contributed to the creation of projects and key technological breakthroughs. It showed that more attention should be paid to international exchange and cooperation in research to promote win-win situation.

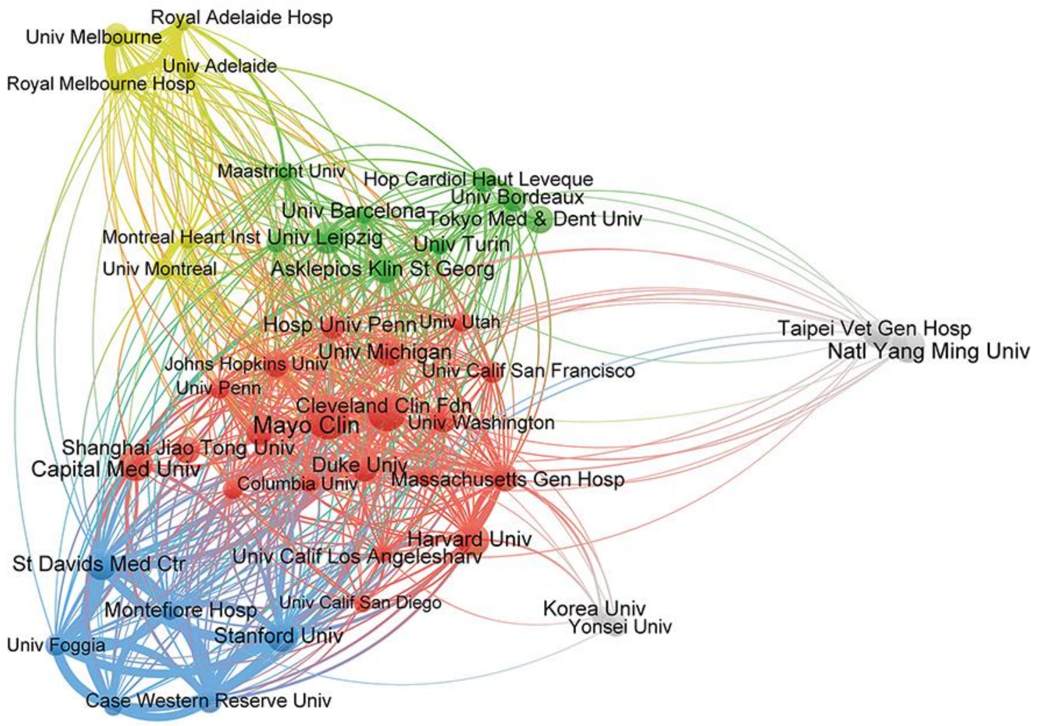

Figure B3 Network map of cooperation between institutions for the catheter ablation and surgical treatment of atrial fibrillation

Co-authorship: authors

A total of 32,983 authors participated in the catheter ablation and surgical treatment of AF study, of whom 3,587 (10.88%) published more than five papers, 1,475 (4.47%) published more than 10 papers, and 506 (1.53%) published more than 20 papers. Table B3 listed the top 20 prolific authors, with a total of 3,015 published papers, accounting for 26.36%. Natale A from St Davis Medical Center in USA published 301 papers, accounting for 2.36%, with a total of 17,897 citations. Followed by Jais P (N=202, 1.59%, Citations =21855) and Haissaguerre M (N=201, 1.58%, Citations =20598) from Hopital Cardiologique du Haut-Leveque. Hindricks G (N=192, 1.51%, Citations =21,703) from Leipzig University (Germany), and Kuck KH (N=180, 1.41%, Citations =8,832) from Asklepios Klinik St Georg (Germany) also published over 180 papers. Figure B4 showed a collaborative network of 64 prolific authors who had published over 70 documents. In total, it was divided into nine different clusters, each of which was replaced by a different color. The top five authors belonged to four different clusters, with Jais P and Haissaguerre M in the same cluster.

Co-authorship analysis can help to analyze the frequency of occurrence of different authors in the same article and reflect the collaborative relationships between authors. Among the top 20 prolific authors, the most published was Natale A of St. Davids Medical Center, with whom Di Biase L was on the same team. In the cluster analysis, both of them were belonged to the same cluster, along with Calkins H from Hopkins University, Marchlinski FE from University of Pennsylvania, and Willems S from University Hospital

Eppendorf. The collaboration between the authors above appearing in the same cluster was much closer. Jais P, Haissaguerre M, Hocini M, and Sanders P from Hopital Cardiologique du Haut-Leveque in France were on the same team. In the cluster analysis, except Sanders P, the other three belong to the same cluster, which may be because Sanders P also work in the Royal Adelaide Hospital in Australia, so they appeared at the intersection of green and pink in Figure B4.

Table B3 Top 20 authors for the catheter ablation and surgical treatment of atrial fibrillation

Rank	Author	Institution	N (%)	Citations
1	Natale A	St Davis Medical Center (USA)	301 (2.36%)	17,897
2	Jais P	Hopital Cardiologique du Haut-Leveque (France)	202 (1.59%)	21,855
3	Haissaguerre M	Hopital Cardiologique du Haut-Leveque (France)	201 (1.58%)	20,598
4	Hindricks G	Leipzig University (Germany)	192 (1.51%)	21,703
5	Kuck KH	Asklepios Klinik St Georg (Germany)	180 (1.41%)	8,832
6	Di Biase L	St Davis Medical Center (USA)	176 (1.38%)	6,218
7	Hocini M	Hopital Cardiologique du Haut-Leveque (France)	164 (1.29%)	18,911
8	Calkins H	Johns Hopkins University (USA)	154 (1.21%)	11,017
9	Sanders P	Hopital Cardiologique du Haut-Leveque (France)	143 (1.12%)	11,663
10	Chen SA	Taipei Veterans General Hospital (Taiwan, China)	133 (1.04%)	5,038
11	Lin YJ	Taipei Veterans General Hospital (Taiwan, China)	125 (0.98%)	3,114
12	Pak HN	Yonsei University (South Korea)	123 (0.97%)	1,811
13	Lo LW	Taipei Veterans General Hospital (Taiwan, China)	119 (0.93%)	2,754
14	Morady F	The University of Michigan (USA)	116 (0.91%)	9,008
15	Kalman JM	Royal Melbourne Hospital (Australia)	116 (0.91%)	5,447
16	Marchlinski FE	Hospital of the University of Pennsylvania (USA)	116 (0.91%)	4,914
17	Ouyang F	Asklepios Klinik St Georg (Germany)	116 (0.91%)	4,490
18	Chang SL	Taipei Veterans General Hospital (Taiwan, China)	116 (0.91%)	2,726
19	Kim YH	Korea University (South Korea)	111 (0.87%)	3,168
20	Willems S	University Hospital Eppendorf (Germany)	111 (0.87%)	2,952

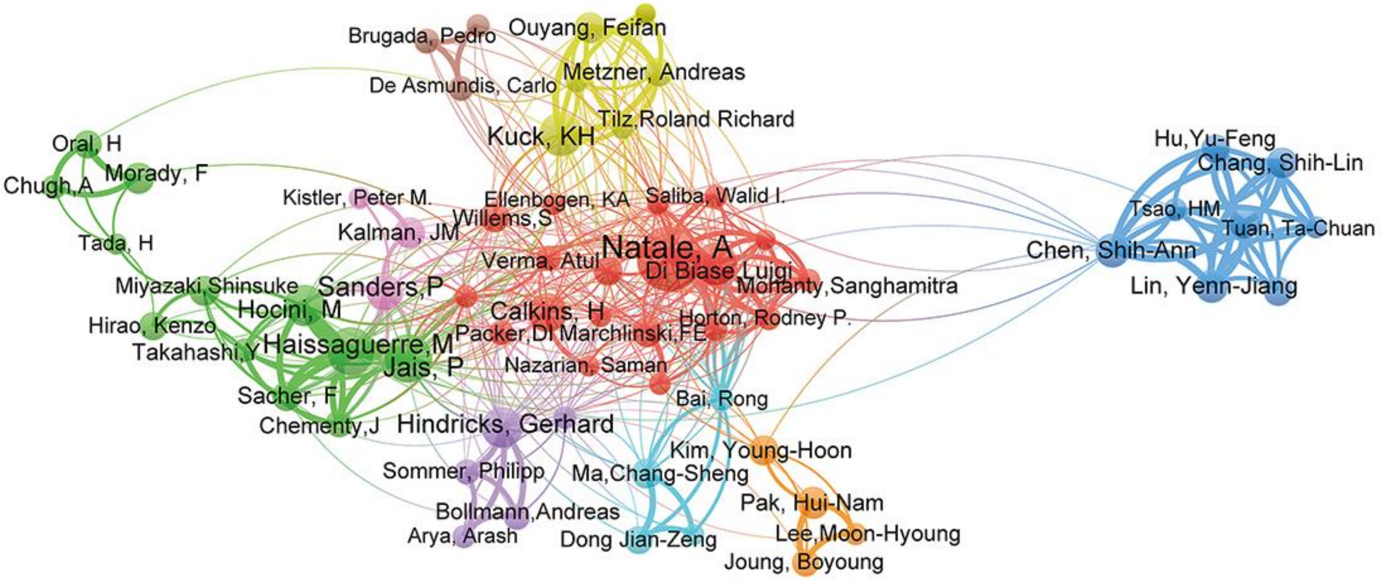

Figure B4 Network map of cooperation between authors for the catheter ablation and surgical treatment of atrial fibrillation
